# Prevalence of pituitary disorders associated with aneurysmal subarachnoid hemorrhage

**DOI:** 10.1186/cc12280

**Published:** 2013-03-19

**Authors:** F Lauzier, I Cote, A Bureau, M Shemilt, A Boutin, L Moore, C Gagnon, AF Turgeon

**Affiliations:** 1 Universite Laval, Quebec, Canada

## Introduction

Pituitary disorders are an often-neglected consequence of aneurysmal subarachnoid hemorrhage (aSAH). We systematically reviewed their prevalence, aiming particularly at studies with low risk of bias.

## Methods

We searched Embase, MEDLINE, The Cochrane Library, Trip Database, references of included studies and narrative reviews. We included cohort studies, cross-sectional studies and RCTs published in any language that tested the integrity of ≥1 pituitary axis in adults with aSAH. Studies including more than 50% of non-aneurismal SAH were excluded. Studies were considered at low risk of bias if the authors defined inclusion/exclusion criteria, avoided voluntary sampling, and tested >90% of included patients with proper detailed diagnostic criteria. Studies testing all pituitary axes were considered as evaluating hypopituitarism, which was defined as the dysfunction of ≥1 axis. We used a Freeman Tukey-type arcsine square-root transformation and pooled prevalences using the DerSimonian-Laird random-effect method. We determined the degree of heterogeneity with *I*^2 ^values.

## Results

Among 12,363 citations, we included 28 studies (1,628 patients). Patients were mostly female (64%) aged 50.5 ± 5.4. Sixteen studies reported the severity of aSAH, 14 reported the procedure for securing the aneurysm and 13 reported the location of aneurysm. Overall, hypopituitarism was observed in 53.4% of patients at short-term (<3 months), 36.5% at mid-term (3 to 12 months) and 34.2% at long-term (>12 months) (Table 1). There was an insufficient number of studies with low risk of bias to perform sensitivity analyses according to study quality.

**Table 1:** 

	Hypopituitarism	GH	ACTH	TSH	Gonadal	ADH
**Short term **						
All studies	**53.4% **(20.5 to 84.7)	**30.4% **(11.5 to 53.2)	**13.2% **(4.4 to 25.1)	**49.2% **(0.1 to 17.7)	**26.1% **(8.8 to 48.0)	**6.3% **(2.7 to 11.0)
*n *(patients)	5 (235)	8 (346)	13 (537)	7 (327)	8 (351)	3 (481)
*I*^2^	96.0%	93.5%	90.2%	89.1%	93.0%	61.1%
Studies of low risk of bias (patients)	2(56)	-	-	2(56)	1(26)	-
**Mid-term **						
All studies	**36.5% **(13.6 to 62.8)	**7.3% **(0.6 to 18.3)	**8.9% **(1.3 to 21.0)	**8.8% **(2.4 to 18.0)	**3.9% **(0.0 to 11.9)	**3.7% **(0.6 to 13.5)
*n *(patients)	4 (109)	6 (158)	7 (208)	5 (176)	6 (178)	2(56)
*I*^2^	85.7%	70.9%	79.4%	59.4%	66.8%	29.0%
Studies with low risk of bias (patients)	1(40)	-	-	1(40)	-	-
**Long-term **						
All studies	**34.2% **(18.7 to 51.4)	**17.1% **(9.4 to 26.4)	**11.2% **(4.8 to 19.4)	**2.3% **(0.4 to 5.3)	**3.2% **(0.7 to 6.8)	**0% **(0 to 1.5)
*n *(patients)	12 (486)	13 (531)	13 (531)	12 (486)	13(351)	3 (142)
*I*^2^	92.5%	82.9%	82.9%	49.0%	62.9%	0%
Studies with low risk of bias (patients)	2(40)	2(40)	2(40)	2(40)	1(10)	-

## Conclusion

The exact prevalence of pituitary disorders following aSAH remains uncertain, mainly due to high heterogeneity and the small number of studies with low risk of bias. However, the prevalence seems to decrease during the recovery phase. The prevalence, risk factors and clinical significance of pituitary disorders in aSAH will require further rigorous evaluation.

